# Assessment of regional pulmonary blood flow using ^68^Ga-DOTA PET

**DOI:** 10.1186/s13550-017-0259-2

**Published:** 2017-01-18

**Authors:** Carlos Velasco, Jesus Mateo, Arnoldo Santos, Adriana Mota-Cobian, Fernando Herranz, Juan Pellico, Ruben A. Mota, Samuel España, Jesus Ruiz-Cabello

**Affiliations:** 1 Centro Nacional de Investigaciones Cardiovasculares Carlos III, C/Melchor Fernández Almagro 3, 28029 Madrid, Spain; 2 CIBER de Enfermedades Respiratorias (CIBERES), C/Monforte de Lemos 3-5, 28029 Madrid, Spain; 3 Department of Anesthesia, Massachusetts General Hospital, Harvard Medical School, 55 Fruit St, 02114 Boston, MA USA; 4 Charles River, Carrer dels Argenters, 7, 08290 Barcelona, Spain

**Keywords:** PET, Pulmonary blood flow, Gallium-68, Fluorescent microspheres

## Abstract

**Background:**

In vivo determination of regional pulmonary blood flow (PBF) is a valuable tool for the evaluation of many lung diseases. In this study, the use of ^68^Ga-DOTA PET for the in vivo quantitative determination of regional PBF is proposed. This methodology was implemented and tested in healthy pigs and validated using fluorescent microspheres. The study was performed on young large white pigs (*n* = 4). To assess the reproducibility and consistency of the method, three PET scans were obtained for each animal. Each radiotracer injection was performed simultaneously to the injection of fluorescent microspheres. PBF images were generated applying a two-compartment exchange model over the dynamic PET images. PET and microspheres values were compared by regression analysis and Bland–Altman plot.

**Results:**

The capability of the proposed technique to produce 3D regional PBF images was demonstrated. The correlation evaluation between ^68^Ga-DOTA PET and microspheres showed a good and significant correlation (*r* = 0.74, *P* < 0.001).

**Conclusions:**

Assessment of PBF with the proposed technique allows combining the high quantitative accuracy of PET imaging with the use of ^68^Ga/^68^Ge generators. Thus, ^68^Ga-DOTA PET emerges as a potential inexpensive method for measuring PBF in clinical settings with an extended use.

**Electronic supplementary material:**

The online version of this article (doi:10.1186/s13550-017-0259-2) contains supplementary material, which is available to authorized users.

## Background

In vivo determination of regional pulmonary blood flow (PBF) is a valuable tool for the evaluation of many lung diseases like chronic obstructive pulmonary disease (COPD), pulmonary hypertension (PH), or pulmonary embolism. Different imaging modalities have been suggested to accomplish this task like magnetic resonance imaging (MRI) [1, 2], scintigraphy [3], single photon emission computed tomography (SPECT) [4], computed tomography (CT) [5], or positron emission tomography (PET) [6, 7].

Determination of blood flow with PET using ^15^O-labeled water is considered the clinical standard [6, 8–10]. However, this technique has very low availability due to the short half-life of ^15^O (2.03 min) that requires an on-site cyclotron for its production [11]. In contrast to cyclotrons, ^68^Ge/^68^Ga generators are cost-effective systems that provide ^68^Ga radionuclide on demand [12]. ^68^Ga has a half-life (68 min) suitable for clinical studies and can be used to label radiopharmaceuticals with low molecular weight. Similar to gadolinium-chelated compounds used in MRI [13], a gallium-68-chelated compound has been suggested by Autio et al. [14] to study blood flow in a preliminary study in rats.

Blood flow measurements with microspheres (MS) is considered the gold standard [15] for the ex vivo validation of different imaging techniques in animals [9, 16, 17]. The MS technique can provide accurate quantitative regional PBF after removing the lung and processing individual tissue samples.

The purpose of this study was to evaluate the feasibility of using a diffusible ^68^Ga-chelated agent, ^68^Ga-DOTA, for in vivo PET imaging of regional PBF and to validate the results using fluorescent MS in pigs.

## Methods

### Synthesis of ^68^Ga-DOTA

^68^Ga (*t*_½_ = 68 min) was available from a ^68^Ge/^68^Ga generator system (ITG Isotope Technologies Garching GmbH, Germany). ^68^Ga was eluted with 4 ml of 0.05 M hydrochloric acid. ^68^Ga was added to a solution containing 200 μg of DOTA (1,4,7,10-Tetraazacyclododecane-1,4,7,10-tetraacetic acid) dissolved in 0.25 M HEPES buffer pH = 4.5. The mixture was heated at 95 °C for 20 min. Finally, 90 μl of NaOH 1 M and 5 ml of NaCl 0.9% were added yielding ^68^Ga-DOTA with a radionuclide purity at radio-HPLC >95% (see Additional file 1).

### Animal preparation

This study was conducted according to the guidelines of the current European Directive and Spanish legislation and approved by the regional ethical committee for animal experimentation. The study was performed on young large white pigs (*n* = 4, mean weight 41.9 ± 5.9 kg) of either sex. To perform the imaging acquisition, animals were sedated with a combination of ketamine 15 mg/kg, xylazine 2 mg/kg, and midazolam 0.5 mg/kg. Animals were kept anesthetized by intravenous infusion of ketamine 4 mg/kg/h and midazolam 0.5 mg/kg/h in saline. The animals were intubated and mechanically ventilated in pressure support ventilation mode with mean fractional concentration of oxygen in inspired gas of 35–40%, tidal volume of 8 ml kg^−1^, and positive end-expiratory pressure ranged from 7 to 9 cm H_2_O. Respiratory rate was ≈18 bpm.

A 7-French pulmonary artery catheter (Swan–Ganz; Edwards Lifesciences World Trade Co. Ltd, Irvine, CA, USA) was inserted through the right external jugular vein using an 8-French introducer sheath (Medtronic, Minneapolis, MN, USA) for continuous pulmonary artery pressure monitoring, MS injection, and blood sample extraction. The Swan–Ganz catheter tip was located in the main pulmonary artery. Additionally, ECG and pulsioximetry were monitored during the entire experiment.

### Image acquisition

**Fig. 1:**
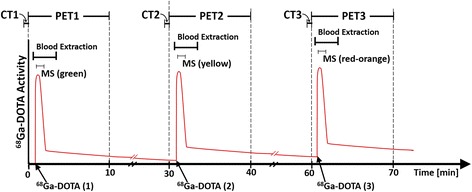
Timeline of the protocol followed for each animal and schematics of the radiotracer accumulation in subsequent scans

PET/CT images were acquired using a Gemini TF-64 scanner (Philips Healthcare, Best, The Netherlands). Animals were placed supine, and three ^68^Ga-DOTA PET scans were obtained under similar physiological conditions for each animal with 30-min interperiod between consecutive scans to allow for physical and biological decay. However, some remaining activity was accumulated in subsequent scans as is schematically shown in Fig. 1. In order to measure the background activity, a 60-s frame was acquired prior to the radiotracer injection and included in the kinetic modeling. No further background corrections were required. Each scan consisted in a low dose CT scan (120 kV, 80 mA) followed by a 10-min list mode PET acquisition in a single bed position covering 18 cm of axial extension. The radiotracer prepared in 7 ml volume was infused with a pump at a rate of 0.2 ml/s (35 s injection) through the marginal ear vein (66 ± 33 MBq). PET scans were started 1 min prior to the radiotracer injection. Each PET acquisition was reconstructed in 41 consecutive frames (1 × 60, 24 × 5, 6 × 10, 3 × 20, 4 × 30, and 3 × 60s). 3D-RAMLA reconstruction algorithm was used in all cases producing images with a voxel size of 4 × 4 × 4 mm^3^.

Each radiotracer injection was performed simultaneously to the injection of 15-μm fluorescent MS (FluoroSpheres®, Molecular probes, Eugene, OR, USA). Each MS injection consisted of 5 × 10^5^ beads diluted into 10 ml of saline containing 0.01% Tween 80. The MS were manually injected over ~60 s directly into the right atrium via the Swan–Ganz catheter. A different MS color was used on each of the three injections provided to the same animal including green (*λ* = 450/480 nm), yellow (*λ* = 515/534 nm), and red-orange (*λ* = 565/580 nm). Immediately before MS dilution, the vial was vigorously vortexed for 15 s, sonicated for 4 min, and vortexed again for additional 15 s. Reference blood samples were drawn from the pulmonary artery through the Swan–Ganz into a heparinized syringe at a rate of 5 ml/min (for 3 min) with an automated syringe pump (Harvard PHD 22/2000 Advance Syringe Pump, Harvard Apparatus), starting 1 min before MS injection. Additionally, another blood sample was taken from the pulmonary artery for hematocrit measurement.

### PET data analysis

**Fig. 2:**
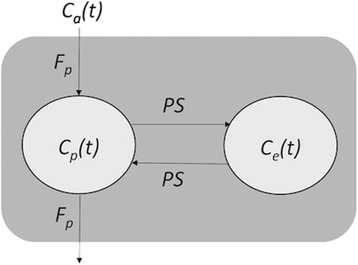
Schematics of the 2CXM model. Radiotracer flows from the arteries to the veins through the capillary bed and extravasates to the interstitial space. *PS* is the permeability-surface product, *F*_*p*_ is the plasma flow, *C*_*a*_ *(t)* is the radiotracer concentration in the arterial plasma, *C*_*e*_ *(t)* is the radiotracer concentration in the interstitial volume, and *C*_*p*_ *(t)* is the radiotracer concentration in the lung capillary bed

PBF derived from MRI typically uses a single compartment model as the acquisition is obtained during one breath-hold and extravasation can be neglected. However, our PET data is acquired in free-breathing during several minutes and extravasation becomes a relevant factor. Therefore, the use of two-tissue compartments was considered to account for the capillary bed and the interstitial volume. In this way, parametric PBF images (PBF_PET_) were generated voxelwise applying a two-compartment exchange model (2CXM) [18] over the dynamic PET images. This model considers the plasma volume of the lung capillary bed as the central compartment which occupies a fractional volume *v*_*p*_, with a tracer concentration *C*_*p*_, and the interstitial volume as the peripheral compartment which occupies a fractional volume *v*_*e*_ with a tracer concentration *C*_*e*_ (see Fig. 2). The exchange rate between the two compartments is described by the permeability-surface product (PS). Thus, tracer concentrations over time (*t*) in both compartments are explained by their mass balance equations (see Eqs. 1 and 2).

1 $$ {v}_p\frac{d{C}_p(t)}{dt}={F}_p{C}_a(t)-{F}_p{C}_p(t)+{\mathrm{PSC}}_e(t)-{\mathrm{PSC}}_p(t) $$

2 $$ {v}_e\frac{d{C}_e(t)}{dt}={\mathrm{PSC}}_p(t)-{\mathrm{PSC}}_e(t) $$

where *C*_*a*_ is the radiotracer concentration in the arterial plasma (arterial input function) and *F*_*p*_ is the pulmonary plasma flow. Tissue tracer concentration is the sum of concentrations in plasma and interstitial space weighted by each particular fractional volume (see Eq. 3).

3 $$ {C}_t(t)={v}_p{C}_p(t)+{v}_e{C}_e(t) $$

This set of differential equations can be solved as the convolution of *C*_*a*_ with the impulse response function (IRF)

4 $$ {C}_t(t)={C}_a\left(t-\delta \right)\otimes \mathrm{I}\mathrm{R}\mathrm{F}(t), $$

5 $$ \mathrm{I}\mathrm{R}\mathrm{F}(t)=\frac{v_e+{v}_p}{v_e{v}_p}\mathrm{P}\mathrm{S}\cdotp {F}_p\left(\frac{e^{-t{E}_{-}}-{e}^{-t{E}_{+}}}{E_{+}-{E}_{-}}\right)+{F}_p\left(\frac{E_{+}{e}^{-t{E}_{+}}-{E}_{-}{e}^{-t{E}_{-}}}{E_{+}+{E}_{-}}\right) $$

where E_+_ and E_−_ are positive, non-zero quantities defined as

6 $$ {E}_{\pm }=\frac{1}{2}\left(\frac{v_e{F}_p+{v}_e\mathrm{P}\mathrm{S}+{v}_p\mathrm{P}\mathrm{S}}{v_e{v}_p} \pm \sqrt{{\left(\frac{v_e{F}_p+{v}_e\mathrm{P}\mathrm{S}+{v}_p\mathrm{P}\mathrm{S}}{v_e{v}_p}\right)}^2-\frac{4{\mathrm{PSF}}_p}{v_e{v}_p}}\right) $$

Therefore, the implemented 2CXM model contains four fit parameters including *v*_*p*_, *v*_*e*_, *F*_*p*_, and PS. The most relevant parameter for our purpose is the pulmonary plasma flow (*F*_*p*_), which can be converted into the pulmonary blood flow (PBF) via the hematocrit value of the lung tissue

7 $$ \mathrm{P}\mathrm{B}\mathrm{F} = \frac{F_p}{1-{H}_T} $$

where *H*_*T*_ represents the hematocrit value.

It can be noticed that the arterial input function employed for the analysis is delayed *δ* seconds with respect to the voxel time-activity curves (TACs) as it was derived from the right ventricle, and there is some delay until it reach the lung capillaries. A delay of 2 s was used. A transit time of 4 s from the right to the left ventricles was observed that further justify the chosen delay value. The same value was used in other studies with ^15^O-labeled water [9]. The delayed AIFs were interpolated to match PET measurement times.

**Fig. 3:**
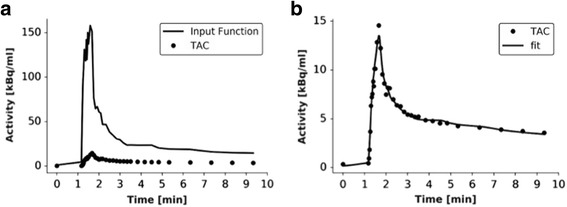
**a** An example of TACs obtained for a large region of the lung (*dots*) and right ventricle (*solid line*). **b** TAC from the lung (*dots*) and fit to a two-compartment exchange model (*solid line*)

The radiotracer flows from the pulmonary arteries to the veins through the capillary bed and extravasates to the interstitial space. Therefore, the radiotracer concentration can be expressed in terms of the plasma concentration in the arterial input, the plasma flow, the plasma volume, the permeability–surface area product, and the interstitial volume. The arterial input function (AIF) (see Fig. 3a) was obtained from the mean voxel value at each time point within a small volume of interest (VOI) drawn inside the right ventricle and was corrected by the pulmonary artery hematocrit to obtain the radiotracer concentration in arterial plasma.

The non-linear fit of the TAC of each voxel was performed by a minimization of the sum of squares using a modified Levenberg–Marquardt method for bound constraints using a Python-coded customized software including SciPy and NumPY libraries. Figure 3b shows and exemplary fit to the average TAC of a large region within the lung. PBF obtained at each voxel was corrected by tissue density. The CT values were used to obtain lung density values (tissue + blood) [19], assuming a linear behavior between CT value and tissue density in the range from air (−1024 HU, *ρ* = 1.225 × 10^−3^ g/cm^3^) to water (0 HU, *ρ* = 1 g/cm^3^) [20, 21]. The blood contribution was subtracted using the plasma fractional volume obtained in the minimization process. Further details are described in Additional file 1.

**Fig. 4:**
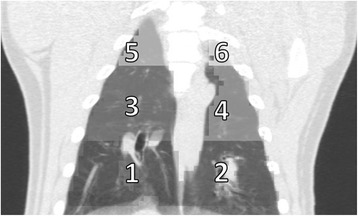
Coronal view of an in vivo CT image indicating the segmentation of the lungs performed for the regional validation against MS. Regions *1*–*2*, *3*–*4*, and *5*–*6* correspond to the basal, medial, and apical zones of the lungs, respectively

On each animal, right and left lungs were segmented separately and each lung was divided into three transversal regions (apical, medial, and basal) with the same axial extension to facilitate regional comparison with the MS measurements (see Fig. 4). The PBF for each region was computed as the average PBF of those voxels with CT values between −925 and 0 HU in order to reject large vessels and airways. A morphological erosion image filtering with 4 mm size was performed over the VOI of the lung to avoid spillover contribution from the heart or liver.

### MS analysis

The MS analysis was based on existing protocols [22] with minor modifications. In brief, the pigs were sacrificed the day after PET scans were performed and the lungs were extracted. The lungs were expanded to try to recover anatomic references and make easier the comparison between zones in PET and excised lungs. To this purpose, the pig was sedated as previously described and euthanized by an intravenous overdose of pentobarbital in supine position while intubated. The animal was mechanically ventilated with similar conditions than the in vivo experiments, the trachea was clamped at the end of inspiration, and the lungs were extracted. An ex vivo CT scan of them was performed to guide the registration between in vivo images and post-mortem validation MS analysis. Afterwards, the lungs were wrapped in a plastic film and frozen at −20 °C. Finally, right and left lungs were separated and individually divided into transversal slices (1–2 cm thickness). The left lung of one of the animals was improperly processed and excluded from the final analysis.

For tissue processing, each lung piece was weighted and immersed in a KOH 4 N solution (5 ml/g of tissue). At the same time, each reference blood sample withdrawn during the experiment was taken to 60 ml with 2% Tween 80, followed by addition of 14 ml of KOH 16 N. All samples were incubated at 37 °C with vigorous shaking during 24–48 h.

After complete tissue digestion, samples were individually filtered with negative pressure filtration. A different filter (Nylon Net Filter 10 μm, Millipore) was used for each sample. Each digested sample was poured onto the filter, and the containing tube was rinsed with ~20 ml of 2% Tween 80 to ensure the collection of all the MS. Next, the filter was rinsed with ~10 ml of buffer rinse solution (KH_2_PO_4_/K_2_HPO_4_) to neutralize the pH of the nylon filter.

Each filter was immersed in 10 ml of cellosolve acetate (2-ethoxyethyl acetate 98%, Sigma) and stored in the dark for 2–3 h, until the complete dissolution of the MS polystyrene cover. The fluorescence of the resultant solution was measured with a Fluorescence Spectrometer (PerkinElmer LS 55) at the emission/excitation wavelengths of 450 nm/480 nm, 515 nm/534 nm, and 565 nm/580 nm for the green, yellow, and red-orange MS, respectively. Both emission and excitation slits were set at 5.0 nm for every reading. Finally, the fluorescence intensities of each sample were used to compute the PBF (PBF_MS_) following the standard MS reference technique [23]. Each sample was assigned to one of the lung six regions segmented in the parametric PBF_PET_ images. Samples out of the field of view were not further analyzed. The PBF of each region was computed adding the fluorescent and weight values of the samples assigned to it.

### Statistic analysis

PBF_PET_ and PBF_MS_ values were directly compared by regression analysis. Regression through the origin (RTO) was performed instead of ordinary least squares (OLS) method as PBF_PET_ values are forced to be non-negative, which produce a positive bias at low PBF. Also, Pearson correlation coefficient was estimated, and Bland–Altman plot was used to assess agreement between PBF_PET_ and PBF_MS_ results. All the measurements were statistically considered as independent events as the MS results showed high variability for measurements repeated on the same animal, and a wide range of PBF values was covered with all the animals. *P* < 0.05 was considered statistically significant.

## Results

### General

Physiological variables were stable during the scan. Heart rate was 100 ± 16 bpm, mean pulmonary artery pressure was always under 25 mmHg, and oxygen saturation was over 98%. A hematocrit value of 0.3, similar to the value used in other perfusion studies with varied techniques, was used for all analyses.

### Parametric PBF maps

**Fig. 5:**
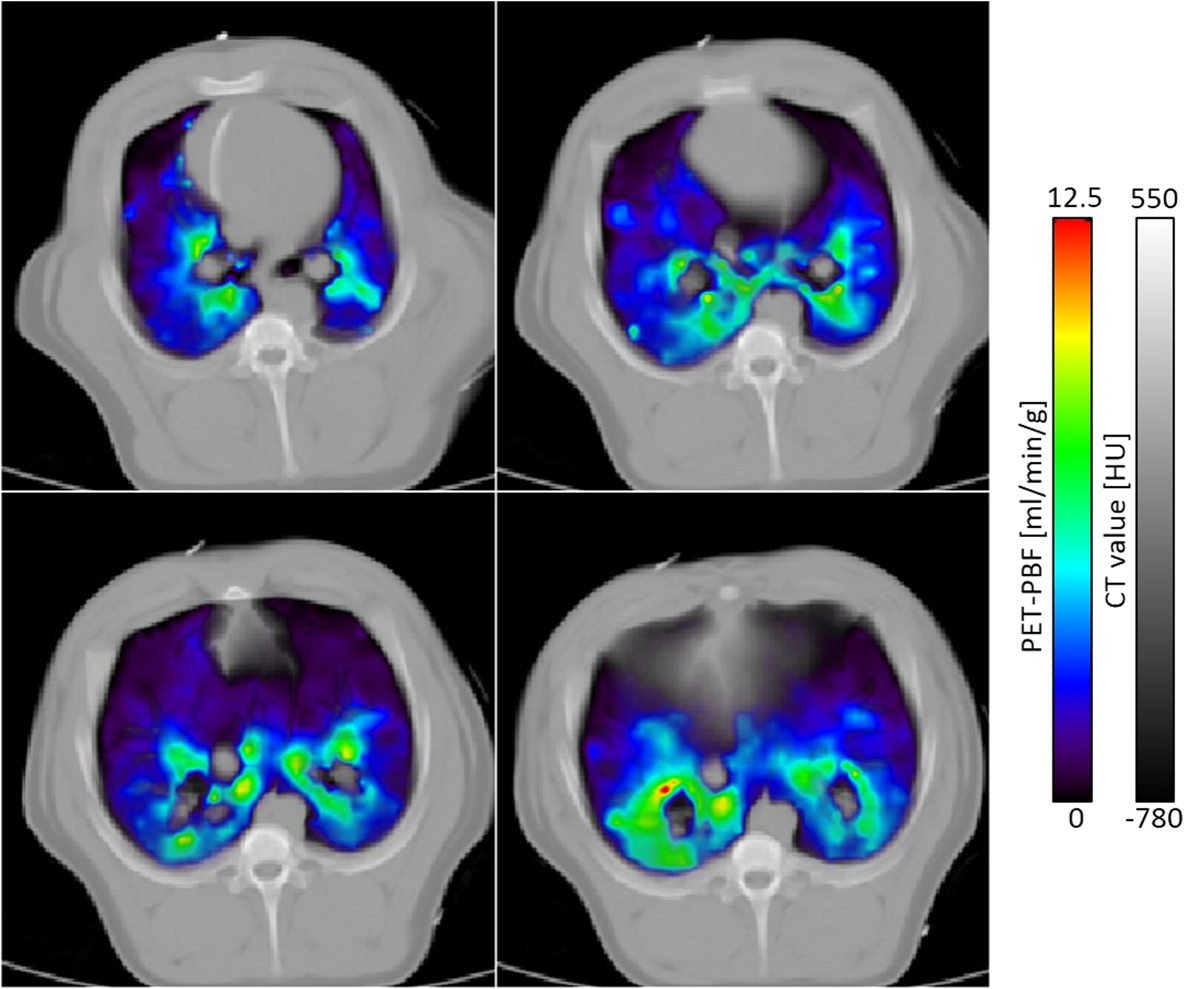
Fused transverse parametric PBF_PET_ and CT images at four different axial locations

Figure 5 shows examples of PBF_PET_ images at different axial slices, demonstrating the capability of this technique to produce 3D regional PBF. The images are fused with their corresponding CT slices for visualization purposes only. PBF values are displayed only for those voxels with CT values in the range from −925 to 0 HU. As expected, the dorsal regions show higher PBF values compared to ventral regions as a consequence of the supine position of the animal during the imaging sessions [24].

### Comparison PBF_PET_ versus PBF_MS_

**Fig. 6:**
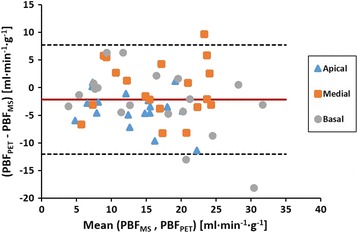
Assessment of agreement between PBF_PET_ and PBF_MS_. Apical (*triangle*), medial (*square*), and basal (*circle*) regions are shown. The *solid lines* represent the mean bias (−2.19 ml min^−1^ g^−1^), and the *dashed lines* represent the 95% confidence interval (mean ± 1.96 SD)

Bland–Altman plot (Fig. 6) shows the evaluation of the agreement between PBF_PET_ and PBF_MS_ including 57 samples. Measured bias was −2.19 ml min^−1^ g^−1^ and limits of agreement (LoA) ranged from −12.06 to 7.69 ml min^−1^ g^−1^. A slight but significant (*P* = 0.0001 for the correlation between PBF_PET_ − PBF_MS_ differences against PBF_PET_ − PBF_MS_ mean) trend to measurement underestimation related with the increase in the PBF value measured was also observed. However, as the effect of this trend was low (*r* ^2^ = 0.0485), no correction was applied to the data.

The MS measurement corresponding to the blood sample of the third scan in pig number 4 showed atypical and extremely low fluorescence values compared to background level. As this can increase the probability of measurement errors, the six points corresponding to this scan were excluded from the analysis. Results of the agreement without the data exclusion lead to a bias and LoA of −3.51 ml min^−1^ g^−1^ and from −16.30 to 9.27, respectively.

**Fig. 7:**
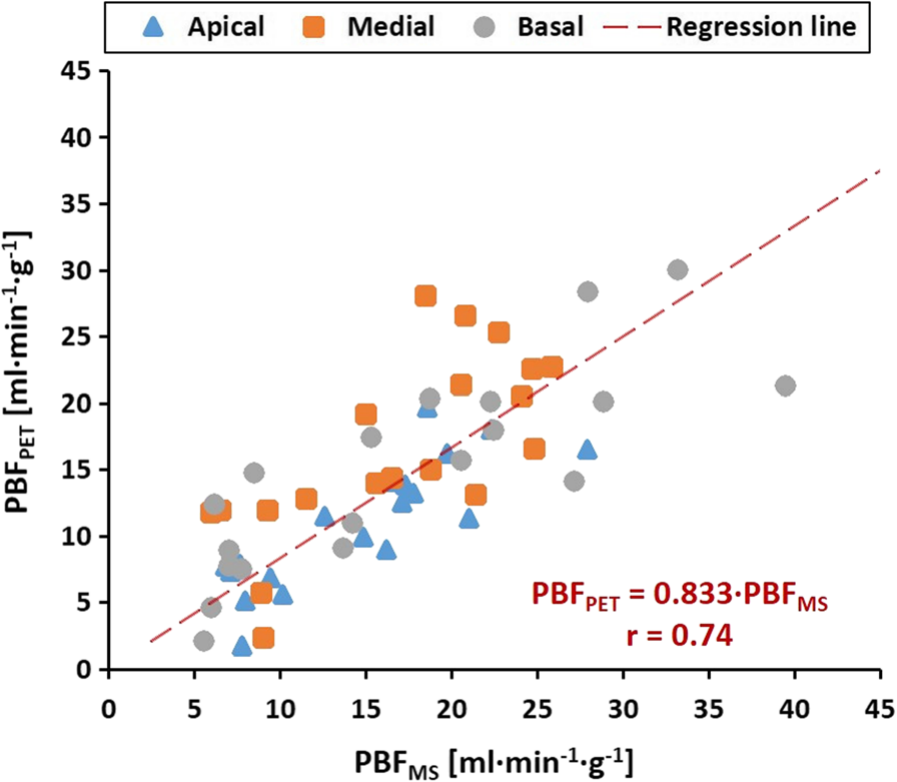
Correlation between PBF_PET_ and PBF_MS_. Apical (*triangle*), medial (*square*), and basal (*circle*) regions are shown. The *dashed line* represents regression line to all the points. Regression line equations are measured in ml min^−1^ g^−1^. Slopes for the regression analysis by lung zones were 0.74, 0.96, and 0.79 for apical, medial, and basal regions, respectively

Evaluation of the correlation between PBF_PET_ and PBF_MS_ including 57 samples is shown in Fig. 7. The methods showed a good and significant correlation (*r* = 0.74, *P* < 0.001) when perfusion across all the measured regions of the lung were evaluated and was maintained when the evaluation was done by lung zones: *r* = 0.84 (*P* < 0.001), *r* = 0.69 (*P* < 0.001), and *r* = 0.76 (*P* < 0.001) for apical, medial, and basal zones, respectively. The goodness of fit worsens (*r* = 0.66, *P* < 0.001 for the entire lung; *r* = 0.76, *P* < 0.001 for the apical; *r* = 0.13, *P* < 0.001 for the medial; and *r* = 0.74, *P* < 0.001 for the basal zone) when the correlation is performed without the exclusion of the six points mentioned before, which is in line with the possibility of measurement errors.

## Discussion

In this study, the use of ^68^Ga-DOTA PET for the in vivo quantitative determination of regional PBF is proposed. The methodology required to perform these studies was implemented and tested in healthy pigs. Finally, the in vivo measurements of regional PBF were validated using fluorescent MS. This is a reliable and easily implementable alternative to dynamic contrast enhanced MRI, where quantification is very challenging due to the non-lineal dependence of signal intensity with the gadolinium-based contrast agent concentration [10]. This is even more important due to the fact that these agents, either linear or macrocyclic, are under investigation for multiple results demonstrating their potential accumulation in various tissues regardless of renal function [25]. Our alternative has a simpler implementation and provides quantitative measures of the regional blood flow compared to recent approaches using dual energy CT imaging [26, 27]. Compared to other PET-based alternatives such as ^15^O-water [7] or dissolved ^13^N_2_ [28] that need an on-site cyclotron, our solution could be potentially more widely available due to the introduction of gallium generators in the field and the approval of different gallium-chelated agents for human use. Our local estimation cost is 100 euros per study including proportional use of the generator, reactive agents, and lab technicians. Compared to other nuclear medicine techniques such as ^99m^Tc-macroaggregated albumin [4], which also reflects true perfusion, SPECT shows lower spatial resolution and longer acquisition times.

We have demonstrated the capability of the proposed technique to obtain in vivo 3D regional PBF. Comparative PBF_PET_ and PBF_MS_ values in dorsal and ventral regions were not obtained due to the difficulty to achieve a good in vivo and ex vivo registration.

The results of this study show potential for ^68^Ga-DOTA radiotracer in clinical practice for measuring PBF. Pulmonary perfusion is an important variable for understanding lung function, especially when it can be described regionally. Such relevancy has allowed that the advances in imaging techniques for lung perfusion and perfusion distribution become an attractive tool for diagnosis and outcomes prediction and evaluation. For example, differences in perfusion patterns measured by dual CT [29, 30] are being proposed for diagnosis of PH and even to differentiate pulmonary arterial hypertension (PAH) from chronic thromboembolic pulmonary hypertension (CTEPH) [31]. Recently, Lau et al. [32] showed using SPECT/CT that pulmonary pre-capillary hypertension showed a redistribution of flow changing the gravidity influence, which appears as a potential tool for disease severity evaluation. Not only PH but also other lung diseases such as COPD have shown alterations in perfusion, which in this disease is related to impaired pulmonary function indexes, appearing also promising for severity evaluation.

The good correlation existing between PBF_PET_ and PBF_MS_ (see Fig. 7) shows high potential for the determination of quantitative regional PBF in vivo and non-invasively using ^68^Ga-DOTA PET. Noteworthy, the *r* value and slope obtained in this study are comparable to those calculated by Schuster et al. [6] (*r* = 0.77, slope = 0.92) and Richard et al. [9] (*r* = 0.79, slope = 0.79) using PET and ^15^O-labeled water. Unfortunately, there is no PBF obtained by CT to compare with our results [26, 27].

The obtained PBF_PET_ values display a small underestimation compared to PBF_MS_ representing only 10% of the maximum PBF obtained. The underestimation could be explained by the difference between the hematocrit of the blood contained in an arteriole or venule of the capillary bed (tube hematocrit H_T_) relative to the hematocrit of blood entering or leaving it (discharge hematocrit H_D_). This difference can be explained by the Fahraeus effect [33] and results in a decreasing hematocrit as blood traverses the microvasculature. In our work, we assumed that H_T_ equals to H_D_, but a proper correction would result in an increment of PBF values as shown in Eq. 8. Pries et al. [34] reported a *H*_*T*_/*H*_*D*_ ratio of 0.65 for *H*_*D*_ = 0.3 at 10-μm diameter capillaries. This correction would increase PBF by 15%.

8 $$ \mathrm{PB}{\mathrm{F}}^{\prime }=\mathrm{P}\mathrm{B}\mathrm{F}\frac{1-{H}_T}{1-{H}_D} $$

It also has to be noticed that Pearson correlation values between PBF_MS_ and PBF_PET_ is limited by the registration inaccuracy between the regions of the in vivo PET scan and the ex vivo MS measurements. A possible improvement for this comparison would be the use of ^68^Ga-labeled MS that could be quantified by a PET scan keeping the animal in the same position as in the ^68^Ga-DOTA scan.

Another source of error can be the delay between the AIF derived from the right ventricle and the arrival of the radiotracer to every tissue voxel [35]. Imprecise delays can produce deviations up to 40% [9]. In this study, we assumed a constant delay of 2 s, but further improvements can be performed in order to include this delay time as a free parameter in the non-linear fit. Finally, the AIF was not corrected by partial volume effect that can have quantitative effects on the results [36].

Although the animals were studied under basal conditions, a wide variability of heart rate and PBF values was obtained. This was probably due to the fact that the animals were mechanically ventilated in pressure support ventilation mode, which required adjusting the anesthesia between consecutive scans in order to maintain the animal stable under mechanical ventilation. However, this effect was transient, and all animals remained stable across each acquisition. In the other hand, such variations allowed us to explore a wider range of perfusion values.

### Limitations

Despite the promising results, this study was performed just in normal animals. However, the main possible applications of this new method are related with the study of diseased states. As pulmonary flow is related with important physiologic processes such as gas exchange and also right ventricle afterload, the ability to track it in a quantitative and reliable way in usual clinical practice could potentially improve disease understanding and patient care. Although non-disease states were studied, PBF was evaluated in a wide range of values. Also, to the best of our knowledge, both the tracer behavior itself and the implemented analysis should remain valid in pathologic conditions. Based on this, we believe our PBF_PET_ using ^68^Ga-DOTA appears as a potential quantitative diagnostic imaging biomarker of endothelial dysfunction in important lung diseases. This, however, still needs to be proven.

Another limitation of our study is the small number of animals (*n* = 4). However, 57 experiments were used for the agreement and correlation studies corresponding to the three scans performed to each animal, and the three transversal regions of each lung. With this, we were able to find acceptable agreement and good and significant correlation between PBF_PET_ and the gold standard measurements. Authors believe that using a larger number of animals could have improved our results through a decrease in measured variability, but it is probably unjustified by the current needs of reducing the number of experimental animals.

Finally, as most of the current implemented methods, ^68^Ga-DOTA PBF_PET_ can offer a regional perfusion estimation, which represents a mean value across the entire respiratory cycle. However, mainly due to the volume and pressure changes within the lungs during the respiration, it is reasonable to expect that the local perfusion be different according to the respiratory phase. In this way, dynamic evaluation of lung perfusion in normal and diseased status could appear as a promissory research field. This aspect could be studied with the proposed technique by applying respiratory synchronization to measured PET data or performing data acquisition during apnea or at the end-of-expiration. However, this was out of the scope of this study.

## Conclusions

In this study, the determination of regional PBF using ^68^Ga-DOTA PET imaging was proposed, implemented, and validated. Assessment of PBF with this technique allows combining the high quantitative accuracy of PET imaging with the much easier access to ^68^Ga/^68^Ge generators compared to existing techniques using H_2_^15^O that require an on-site cyclotron. In this way, ^68^Ga-DOTA PET appears as a potential method for measuring PBF in clinical settings with an extended use.

## Additional file

Additional file 1: Supplementary information including the radiotracer purity assessment by HPLC and *PBF* units conversion. (DOCX 59 KB)

## Abbreviations

2CXM: Two-compartment exchange model
AIF: Arterial input function
*C*_*a*_: Radiotracer concentration in the arterial plasma
*C*_*e*_: Tracer concentration in the peripheral compartment
COPD: Chronic obstructive pulmonary disease
*C*_*p*_: Tracer concentration in the central compartment
CT: Computed tomography
CTEPH: Chronic thromboembolic pulmonary hypertension
DOTA: 1,4,7,10-Tetraazacyclododecane-1,4,7,10-tetraacetic acid
ECG: Electrocardiogram
*F*_*p*_: Pulmonary plasma flow
*H*_*D*_: Discharge hematocrit
*H*_*T*_: Tube hematocrit
HU: Hounsfield unit
IRF: Impulse response function
LoA: Limits of agreement
MRI: Magnetic resonance imaging
MS: Microspheres
OLS: Ordinary least squares
PAH: Pulmonary arterial hypertension
PBF: Pulmonary blood flow
PBF_MS_: PBF value obtained with microspheres
PBF_PET_: PBF value obtained with PET
PET: Positron emission tomography
PH: Pulmonary hypertension
PS: Permeability-surface product
RTO: Regression through origin
SPECT: Single positron emission computed tomography
TAC: Time-activity curve
*v*_*e*_: Interstitial fractional volume
VOI: Volume of interest
*v*_*p*_: Plasma fractional volume
