# Supplementary material for: Assessment of regional pulmonary blood flow using 68Ga-DOTA PET
Source: EJNMMI Res. 2017 Jan 18;7:7. doi: 10.1186/s13550-017-0259-2 (PMC5241570; doi:10.1186/s13550-017-0259-2)
Supplement: Additional file 1: — Supplementary information including the radiotracer purity assessment by HPLC and PBF units conversion. (DOCX 59 KB) [file 13550_2017_259_MOESM1_ESM.docx]

Synthesis of ^68^Ga-DOTA

The radiochemical purity of ^68^Ga-DOTA was determined by reversed-phase radio-HPLC (Knauer smart series coupled with Gabi Raytest® detector). The HPLC conditions for ^68^Ga-DOTA were as follows: flow 3 ml/min, A = 2.5 mM trifluoroacetic acid (TFA), B = acetonitrile (MeCN). Linear A/B gradient: 0–5 min 100/0, 5–10 min from 100/0 to 60/40. Free ^68^Ga^+3^ were pre-injected under the same conditions comparing the retention times. After the reaction with DOTA the retention time changed from 3.99 min to 3.32 min, showing a radiochemical purity > 95 % (see figure S1).


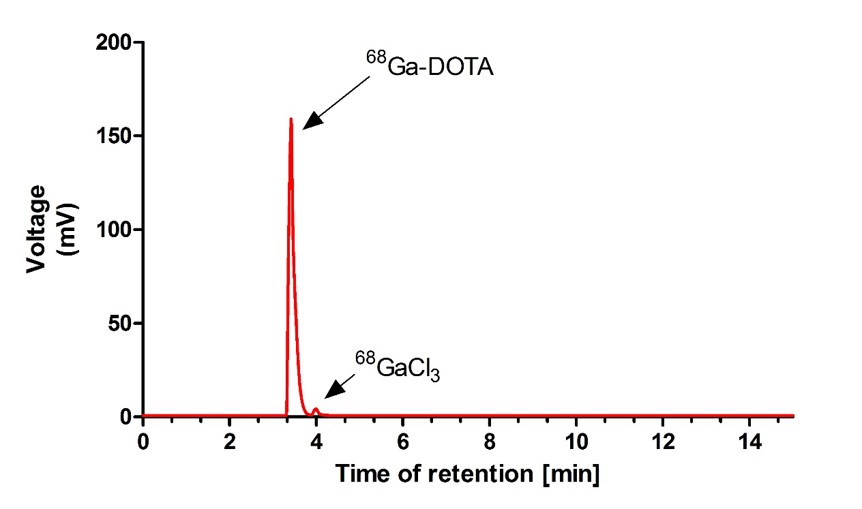


Figure S1: ^68^Ga-DOTA peak purity assessed with HPLC shows a purity > 95 %.

PET data analysis

In equation 7 of the main text of the manuscript, the *PBF* is obtained in units of volume of blood per time and volume of tissue (ml min^-1^ ml^-1^) but, in order to compare with the microspheres (MS) results, *PBF* must be expressed as volume of blood per time and mass of tissue (ml min^-1^ g^-1^). To account for this, *PBF* value was corrected by density and blood fraction. The CT values were used to obtain lung density values (tissue + blood)^1^ assuming a linear behavior between CT value and tissue density in the range from air (-1024 HU, *ρ* ~ 0 g/cm^3^) to water (0 HU, *ρ* = 1 g/cm^3^)^2,3^. The blood contribution was subtracted using the plasma fractional volume (*v_p_*) obtained in the minimization process (see equations S2 and S3).

|  | $PBF\left[ ml\cdot\min^{-1}\cdot g^{-1} \right]= \frac{PBF\left[ ml\cdot\min^{-1}\cdot{ml}^{-1} \right]}{\rho_{lung}-\frac{v_{p}}{1-H_{T}}\rho_{blood}},$ | (S1) |
| --- | --- | --- |

where a blood density (*ρ_blood_*) of 1 g/cm^3^ was used and lung density (*ρ_lung_*) was computed in a pixel by pixel basis as

|  | $\rho_{lung}= \frac{1024+CT_{i}}{1024},$ | (S2) |
| --- | --- | --- |

where *CT_i_* is the CT value (HU) measured at voxel *i*.

REFERENCES

1. Holman B, Cuplov V, Millner L, et al. Improved correction for the tissue fraction effect in lung PET/CT imaging. *Phys Med Biol*. 2015;60(18):7387-7402.

2. Saw CB, Loper A, Komanduri K, Combine T, Huq S, Scicutella C. Determination of CT-to-density conversion relationship for image-based treatment planning systems. *Med Dosim*. 2005;30(3):145—148.

3. Constantinou C, Harrington JC, DeWerd LA. An electron density calibration phantom for CT‐based treatment planning computers. *Med Phys*. 1992;19(2).
